# Report of a Case

**Published:** 1892-08

**Authors:** R. R. Kime

**Affiliations:** Atlanta, Ga., Lecturer on Gynecology Southern Medical College


### REPORT OF A CASE.

BY R. R. KIME, M. D., ATLANTA, GA.,
Lecturer on Gynecology Southern Medical College.

Mrs. R., age 34, widow four years. Had two children, youngest 8 years old. No miscarriages. First flow at fourteen years of age, regular but scant, and for last two years very little, not lasting but one day. Severe pains at flow, before and after; much worse last two or three years; leucorrhœa. No special difficulty at confinements. "Rigors of pain," as she expressed it, at flows and often between times, especially when food did not digest well. Has taken a great deal of soda for "sour stomach;" also calcined magnesia. Pain principally in lower part of bowels, mostly on right side. Tenderness constant; worse at times and most marked on right side. Had rupture on same side at 12 years of age, for which she wore a truss eighteen months, and recovered. Bowels always constipated except within last twelve months. Dates trouble as commencing four or five years back; worse since an attack of la grippe in Janu.ry, 1891. Confined to bed since last December, not able to do any work for last eight or ten months. Been under treatment of different physicians for the last two or three years.

Examination revealed lacerated cervix, uterus enlarged. Right ovary enlarged and prolapsed, but on account of excessive tenderness and pelvic deposits, could not map out tubes and left ovary.

Under local vaginal applications of iodine, boro-glyceride and glycerine tampons, with hot water vaginal douches, the deposits seemed to clear up to some extent, but the tenderness prevented a satisfactory examination without anæsthetic.

From history of case, physical condition, almost constant elevation of temperature for some months, and amount of suffering, an abdominal section was decided upon by Dr. J. C. Avary and myself, without further examination.

A severe attack, May 3d, of intercostal neuralgia, followed by sick stomach and accumulation of gas in bowels, caused postponing of operation to May 10th. Evening before operation, temperature 100 degs. Had not been clear of fever at any time since arrival, April 28th.

Physicians present at operation: Drs. Avary, Elkin, Childs and C. Evans Johnson.

The right tube and ovary were brought up and removed with very little difficulty. Left ovary could not be reached until incision was enlarged to admit all the fingers of one hand. In separating and bringing it up, encountered a hard nodule about size of normal ovary, which, on inspection, seemed to be a deposit in wall of descending colon, and by the advice of Drs. Elkin and Avary was dropped back and let alone.

Abdominal incision closed with silk worm gut. Within eighteen hours after operation, 1-6 and 1-10 grains hypodermics of morphia were used to relieve intense pain. Bowels moved second day by enemata of salts, quinine and glycerine, as she could retain nothing by stomach. Highest temperature three days after operation, 101 1-2 degs., due to getting out of humor, bowels not moving sufficiently and taking some nourishment that did not agree with her, after which diet was limited and bowels were kept moving each day by enemata and calomel triturates, which acted promptly in relieving pain and reducing temperature.

Removed stitches on 9th day. Union perfect, not a drop of pus. Sat up on the 15th day; returned home on the 19th day—a distance of 100 miles—as she said, feeling better than she had in two years. Suffered very little pain returning home.

*Remarks.* Very little changes in tubes and left ovary. Right ovary contained a cyst holding one ounce or more of bloody serum; examined microscopically by Dr Childs. We decided it to be an endothelioma of the corpus luteum changing to a hematoma.

The literature on this condition of the ovary seems to be very scarce, while I am inclined to believe that diseased conditions of the corpus luteum are of no uncommon occurrence.

The most extensive article I know of on this subject is the one by Francis Foerster, in *Amer. Jour. of Obst. and Diseases of Women*, May, 1892. He states that Dr. M. D. Jones designates these conditions of the corpus luteum as "endothelioma changing to angioma and hematoma, and gyroma as a prestage of endothelioma." Foerster states, "It indeed appears that what was previously called a corpus luteum is invariably an endothelioma. This formation in some instances is pathological; as such it is always morbid when changes take place toward the formation of hematoma. The physiological corpus luteum, or endothelioma of pregnancy represents the only exception." Dr. Foerster bases his conclusions on an analysis, clinical and microscopical, of twenty-five cases of ovaries removed by abdominal section.

Pozzi, in his late work, devotes but one page to cysts of the corpus luteum, and claims that Rokitansky was the first to describe this form of cyst, and believed that the corpus luteum of pregnancy only could be transformed into a cyst.

Later investigations have demonstrated that cysts gyroma, endothelioma and hematoma may develop in the corpus luteum of menstruation. None of them attain a very large size, but are often very painful, and produce general nervous manifestations. It will be a question of the future to decide the necessity of the removal of the entire ovary in these conditions, or whether the diseased structure may simply be removed, and leave behind the healthy portion of the ovary.

It is here that the general practitioner should recognize that there are conditions of the ovary outside of pus formation and the ovarian cyst proper, that demand abdominal section and removal of the offending member for relief of patient. These diseased conditions of the corpus luteum, causing such intense suffering and invalidism, are very difficult to recognize by physical examination.

It is just such cases that the general practitioner often treats for months or years without relief—the only result often being to aggravate the condition augmenting the inflammatory condition and increasing the adhesion, thereby rendering the operation more difficult when resorted to as a last chance of relief. There is also another danger which I do not think is imaginary, and that is rupture of these cysts or hematomæ of the corpus luteum, with escape of contents, setting up a pelvic peritonitis; also, in the treatment of these cases, the physician should be cautious and not use too much force or pressure in replacing ovary or uterus, or breaking up adhesions, for fear of rupture as above mentioned.

When a general practitioner has a case of this character, which ordinary means fail to relieve, the case should be referred to one doing special work in that line, to decide as to the advisability of operative measures.

It is an injustice to the patient to keep her a sufferer and an invalid year after year with temporizing measures, when by a comparatively safe operation, she can be restored to health and usefulness.

